# Latest Advances on Bacterial Cellulose-Based Antibacterial Materials as Wound Dressings

**DOI:** 10.3389/fbioe.2020.593768

**Published:** 2020-11-23

**Authors:** Lu Zheng, Shanshan Li, Jiwen Luo, Xiaoying Wang

**Affiliations:** ^1^State Key Laboratory of Pulp and Paper Engineering, South China University of Technology, Guangzhou, China; ^2^Key Laboratory of Theoretical Chemistry of Environment, Ministry of Education, School of Chemistry and Environment, South China Normal University, Guangzhou, China

**Keywords:** bacterial cellulose, wound dressings, infection, antibacterial activity, sustained release

## Abstract

At present, there are various wound dressings that can protect the wound from further injury or isolate the external environment in wound treatment. Whereas, infection and slow self-healing still exist in wound healing process. Therefore, it is urgent to develop an ideal wound dressing with good biocompatibility and strong antibacterial activity to promote wound healing. Bacterial cellulose is a kind of promising biopolymer because it can control wound exudate and provide a moist environment for wound healing. However, the lack of antibacterial activity limits its application. In this paper, the advantages of bacterial cellulose as wound dressings were introduced, and the preparation and research progress of bacterial cellulose-based antibacterial composites in recent years were reviewed, including adding antibiotics, combining with inorganic antibacterial agents or organic antibacterial agents. Finally, the existing problems and future development direction of bacterial cellulose-based antibacterial wound dressings were discussed.

## Introduction

**FIGURE 1:**
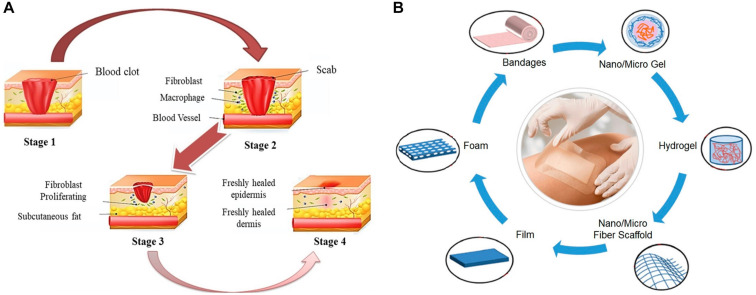
**(A)** Wound self-healing process (Moeini et al., 2020). **(B)** Different forms of wound dressings (Teixeira et al., 2020).

Human skin covers the whole body surface, which is the barrier between human body and external environment, and plays an important role in preventing pathogens from entering human body (Byrd et al., 2018). In addition, the skin can regulate a variety of body functions, has effects on protection, excretion, temperature regulation and external stimulus perception, and also supports blood vessels and nerves (Yildirimer et al., 2012). Wound repair is a dynamic process, which mainly consists of several stages (Figure 1A). The first reaction after injury is hemostasis, which occurs at the site of blood loss in the wound (Sinno and Prakash, 2013). The second stage is inflammation lasting from 24 h to 4–6 days, which begins with releasing proteolytic enzymes and proinflammatory cytokines from invasive immune cells into the wound area, and these inflammatory cells produce reactive oxygen species (ROS) to protect the organism from bacterial infection (Thomason et al., 2018). At this stage, all foreign bodies and tissue debris are removed from the wound bed by neutrophils and macrophages, thus preventing infection (Minutti et al., 2017). Furthermore, the release of cytokines and enzymes stimulate the growth of fibroblasts and myofibroblasts, and wound exudate ensures the necessary moisture for healing (Das and Baker, 2016; Suarato et al., 2018). The third stage is the proliferation stage, in which new granulation tissue forms and grows in the wound area to form the new extracellular matrix. The final stage of healing is remodeling. At this stage, the matrix composition changes and collagen III is replaced by collagen I, which leads to the increased tensile strength of new tissues (Bielefeld et al., 2013; Sinno and Prakash, 2013; Das and Baker, 2016). Skin wound repair is a complex physiological process, which is often affected by uncertain factors. Self-healing of wound is slow and susceptible to external infections, hence appropriate wound dressing is needed to promote and guide the healing process.

Optimal dressings are defined as being able to maintain high humidity at the wound site, remove excess exudates, have non-toxicity and non-allergy reaction, allow oxygen exchange, prevent microbial invasion, be comfortable, and cost-effective (Jannesari et al., 2011; Dart et al., 2019; Rezvani Ghomi et al., 2019; Felgueiras et al., 2020). Both natural and synthetic polymers can be used to prepare wound dressings, while natural polymers are widely used in wound dressings due to their biocompatibility, biodegradability, high bionic level and physicochemical properties. In particular, biopolymer based wound dressings, whose degradation rate should be synchronized with the wound healing process, should ensure the effective release of active substances at the same time (Suarato et al., 2018). Natural polymer materials that have been studied as wound dressing for the moment include agar, sodium alginate, hyaluronic acid, chitin, chitosan, carrageenan, cellulose, pectin, starch, collagen and so on. Moreover, different forms of wound dressings can be prepared, including bandages, hydrogel, film, sponge, foam, nanofiber mats, etc (Ambekar and Kandasubramanian, 2019; Teixeira et al., 2020; Figure 1B). On account of that wound dressings are the barrier to protect wounds, it is particularly significant to develop suitable wound dressings. The ideal wound dressings should promote healing and bring the least inconvenience to patients. At the same time, they can remove excessive exudate, improve autolysis debridement, and keep enough water for healing. In addition, wound dressings should also have certain applicability, flexibility, stability, barrier property, biodegradability, certain viscosity and be easy to remove, so as to speed up the healing speed and reduce the chance of infection (Boateng and Catanzano, 2015; Suarato et al., 2018).

Wound repair is a physiological process affected by many factors, and its complexity often leads to some uncertainties, such as slow wound healing, secondary infection, and inflammation (Au-Crane et al., 2020). Therefore, it is very important to choose appropriate wound dressing to promote and guide the healing process. Bacterial cellulose (BC), as a new type of wound dressing material, has attracted more and more attention. Various species of bacteria can produce cellulose in a biosynthesis pathway that involves the secretion of polysaccharides produced from carbon sources (Fernandes et al., 2020). This nanostructured cellulose has good physicochemical and mechanical properties, outstanding biocompatibility and biodegradability (Sulaeva et al., 2015). Compared with plant-derived cellulose, BC has high purity, simple purification process and three-dimensional (3D) network structure similar to extracellular matrix, so it is widely studied in tissue engineering and reconstruction of damaged tissues, wound healing and vascular regeneration (Li Z. et al., 2016; Frone et al., 2020; Pang et al., 2020). In addition, BC has the potential to be applied to various fields such as drug delivery, bioprinting, implants, artificial organs, and others (Ul-Islam et al., 2015; Ullah et al., 2016; Yang et al., 2018). BC has many excellent properties for wound healing, but the lack of antibacterial activity limits its application in wound dressings (Wei et al., 2011). The antibacterial activity of wound dressings plays an important role in anti-infection and promoting wound healing during wound treatment.

**FIGURE 2:**
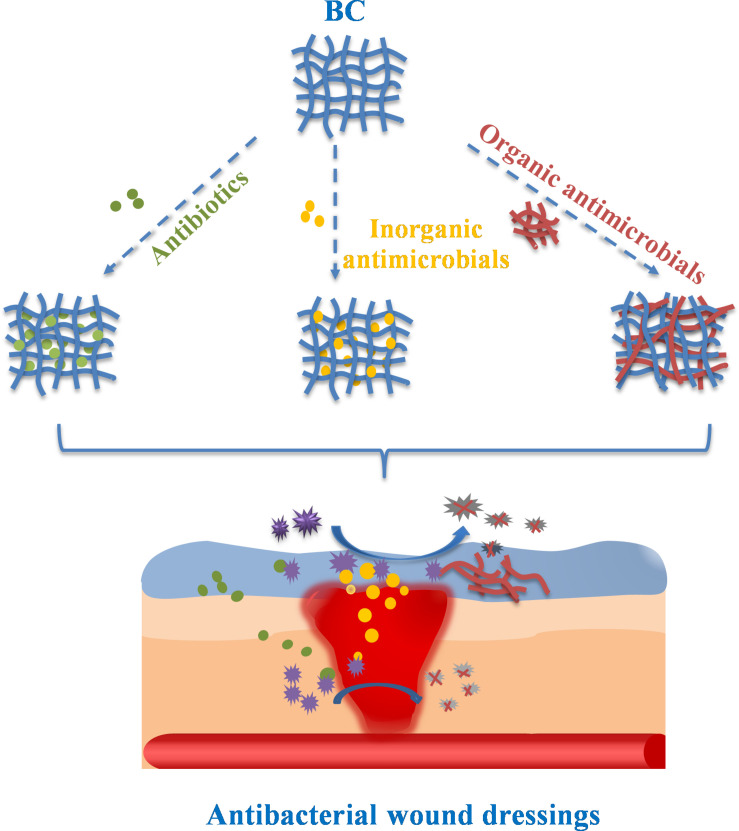
The preparation and slow release behavior of BC-based antibacterial wound dressings.

In order to endow BC with antibacterial properties, many studies have prepared BC-based composite materials with antibacterial function as wound dressing. The high porosity and surface area of BC allow the introduction and release of antimicrobial agents, drugs and other biological functional materials (Buruaga-Ramiro et al., 2020). This paper reviews the preparation methods and slow release behavior of BC-based antibacterial materials for wound dressings, as shown in Figure 2.

## Bacterial Cellulose

### Brief Description

**FIGURE 3:**
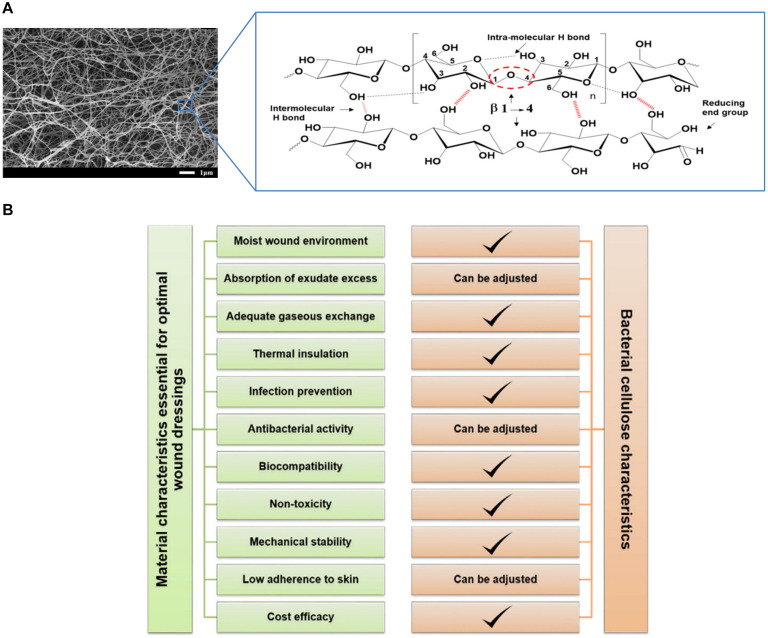
**(A)** General introduction of BC tissue structure (Shao et al., 2016a; Tayeb et al., 2018). **(B)** An overview of BC characteristics with respect to the general requirements for wound dressing materials (Sulaeva et al., 2015).

Bacterial cellulose is a natural polymer produced by bacterial strains with 3D network structure composed of cellulose chains, and the network can be filled with a large amount of liquid to support tissue regeneration (Sulaeva et al., 2015; Figure 3A). The production of BC dates back to 1886 (Brown, 1886). Various species of bacteria can produce cellulose in a biosynthesis pathway that involves the secretion of polysaccharides produced from carbon sources (Fernandes et al., 2020). Microorganisms that synthesize BC include *Acetobacter*, *Agrobacterium*, *Rhizobium*, *Sarcina*, etc., and carbon sources could be glucose, fructose, maltitol, sucralose, lactose, etc. Agricultural corn stalk, nutshell, other agricultural or industrial waste also could be carbon sources (Cheng et al., 2017; Dórame-Miranda et al., 2019; Ul-Islam et al., 2020). Besides, BC can also be synthesized in a cell-free system which does not have a complete cell structure but retains a biosynthetic cell extract. Ullah et al. (2015) hypothesized a synthetic pathway for bio-cellulose synthesis in the cell-free system, which can overcome some limitations of cellulose-producing cells and offers a wider scope for synthesizing cellulose composites with bactericidal elements. The production and yield of BC are influenced by carbon source concentration, species and other medium components. The majority of low-cost culture media have the potential to produce BC on an industrial scale (Velásquez-Riaño and Bojacá, 2017). Optimization strategies (conventional or statistical) have become relevant for the cost-effective production of BC (Fernandes et al., 2020).

Bacterial cellulose is known for its many desirable properties, such as sustainability, biocompatibility, biodegradability, extensive chemical modification capability, and high surface area. Although BC and plant-derived cellulose all have the same chemical structure of linear β-1,4-glucan chains, there are still many differences in physical properties between them (de Amorim et al., 2020). Plant-derived cellulose chains are closely related to hemicellulose, lignin, etc., while BC contains no other polymers so that it has high purity and simple purification (Klemm et al., 2011). The continuous spinning of cellulose ribbons by bacteria leads to the formation of 3D network structure of nanofibers stabilized by hydrogen bonds (Luo et al., 2014). The special fiber network structure endows BC with unique mechanical properties like high crystallinity and high Young’s modulus, the highest of all two-dimensional organic materials (Dayal and Catchmark, 2016). As a result of the high aspect ratio of the fiber, BC with a high specific surface area can provide a liquid loading capacity of up to 99 wt.%. In the presence of water, about 90% of water molecules are tightly bound to a large number of hydroxyl groups in cellulose molecules (Bashari et al., 2018). Compared with plant-derived cellulose, BC has larger specific surface area. The water absorption of BC is 30% higher than that of cotton yarn, and the drying time is prolonged by 33% (Meftahi et al., 2010).

### Bacterial Cellulose as Wound Dressings

The common biomass materials for wound dressings include chitosan, gelatin, hyaluronic acid, etc (Wu J. et al., 2018; İnal and Mülazımoğlu, 2019; Graça et al., 2020). These materials have good biocompatibility, degradability, mechanical properties, etc., but BC has unique advantage in the application of wound dressings for it has more excellent properties at the same time. The use of BC as a wound healing material is determined by its unique characteristics, including high tensile strength, good flexibility, strong water holding capacity, significant permeability to gas and liquid, great compatibility with living tissues and so on. In terms of biocompatibility, BC itself has no cytotoxicity and no toxic substances are introduced in the production process, and there are no adverse reactions produced when BC contacts with human body in short-term or long-term because of its histocompatibility and blood compatibility. These unique properties of BC are closely related to its source, structure and functional characteristics, which have a certain impact on wound healing (Table 1). In addition, pure BC can be modified specifically to meet all the necessary functional requirements as a wound dressing (Sulaeva et al., 2015; Figure 3B).

**TABLE 1:** The relation among BC structures, BC properties, and wound healing.

**BC structures**	**BC properties**	**Effects on wound healing**	**References**
Composition of glucose monomer	Biocompatibility and biodegradability	have good compatibility with living tissue and avoid allergic reactions and foreign body rejection	Mohamad et al., 2017; Rühs et al., 2018; Torgbo and Sukyai, 2020
3D network structure	Mechanical stability and certain permeability to liquids and gases	1. Allow the absorption and evaporation of exudate and the oxygen exchange, and protect the wound from infection	
Fiber with high aspect ratio and high specific surface area	High tensile strength, good flexibility and high liquid loading capacity	2. Have stable mechanical properties, and easy to remove from the wound to avoid secondary injuries	Meftahi et al., 2010; Klemm et al., 2011; Luo et al., 2014; Dayal and Catchmark, 2016; Bashari et al., 2018; Portela et al., 2019; Nuutila and Eriksson, 2020
Abundant hydroxyl group	Strong hydrophilicity and water retention	3. Provide a warm and moist micro-environment for the wound, which is conducive to wound healing	

The normal progress of wound healing mechanism may be destroyed by many factors, resulting in the prolongation of healing time. In this case, the demand for dressings has risen to a new level that requires them to actively participate in the complex wound repair process. BC, as one of the promising wound dressing materials, can control wound exudate and provide moist environment for wound healing (Nuutila and Eriksson, 2020), but it lacks antibacterial activity that limits its application. Complications can easily occur in chronic wounds where severe physiological changes or tumorigenesis may happen, which can lead to excessive production of exudates containing high levels of tissue-destructive proteases and contamination of wounds by foreign bodies (e.g., bacteria) because of inflammatory reactions (Lindsay et al., 2017; Ruf et al., 2017). For example, more than 50% of diabetic chronic ulcers are infected, and the infection is one of the most common and serious sequelae during wound healing and also the major factor in delayed healing (Alavi et al., 2014). Furthermore, due to the adverse effect of the patients’ immune system and the extensive destruction of physical skin barrier, which cannot prevent the invasion of bacteria, the problem of microorganism colonization in the wounds of burn patients is particularly serious (Oryan et al., 2017). Therefore, the development of wound dressings with antibacterial activity is particularly important for reducing bacterial infection and promoting wound healing.

## Bacterial Cellulose-Based Antibacterial Wound Dressings

In order to prepare BC-based composites with good antibacterial properties as wound dressings, three methods can be used: adding antibiotics, combining with inorganic antibacterial agents, and combining with organic antibacterial agents. Adding antibiotics is the most commonly used method, which has been widely used in clinical application. Inorganic antibacterial agents have excellent antibacterial properties and have a great application prospect in the field of biomedicine. Natural organic antibacterial substances have attracted wide attention due to their good biocompatibility and biodegradability.

### Addition of Antibiotics

**FIGURE 4:**
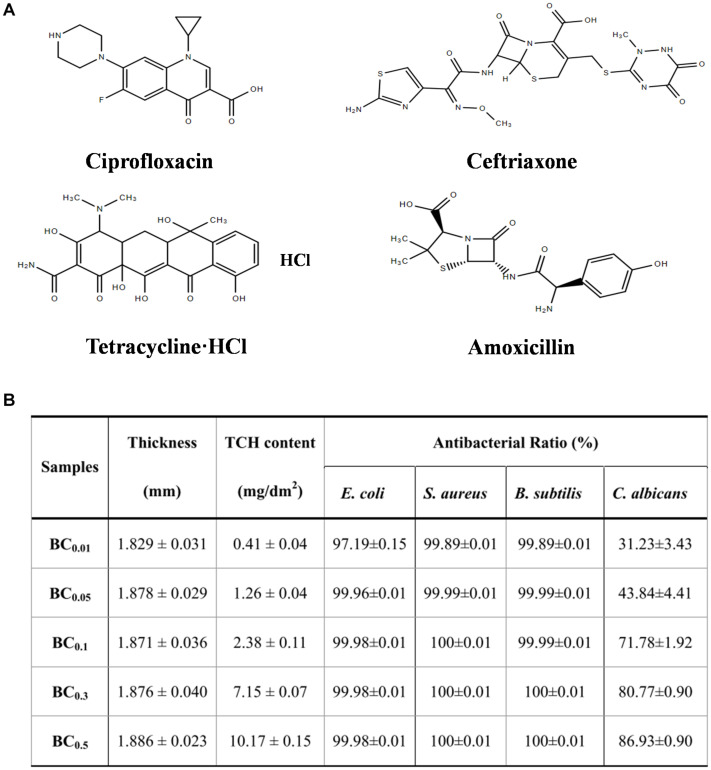
**(A)** Structural formula of four common antibiotics. **(B)** Antibacterial properties of BC-TCH composite films with different TCH content (Shao et al., 2016a).

The antibacterial activity of BC-based wound dressings is often achieved by adding antibiotics. The most commonly used antibiotics are ciprofloxacin, ceftriaxone, tetracycline hydrochloride (TCH), amoxicillin and so on (Figure 4A). Chemical modification and the incorporation of antibiotics can further increase the potential of BC as a biomedical material. BC is composed of β-1,4-glucose, where there are three active hydroxyl groups in C2, C3, and C6 of glucopyranosyl ring. BC can be easily modified at these sites by oxidation, esterification or etherification to introduce new functional groups. Immersion method is the common method of combining BC with antibiotics, which is simple and easy to operate but may cause uneven distribution of antibiotics. For example, vancomycin and ciprofloxacin can be incorporated into bacterial nanocellulose (BNC) or modified BNC to provide bioactivity for wound dressing and tissue engineering scaffold (Vismara et al., 2019). The BC composite materials containing amikacin and ceftriaxone were prepared by immersing the dried BC films in different concentrations of antibiotics solution. And the composites have obvious antibacterial activity against *Escherichia coli*, *Pseudomonas aeruginosa*, *Streptococcus pneumoniae*, and *Staphylococcus aureus*, so they are expected to be used as wound dressings (Volova et al., 2018). Moreover, it was observed that the bilayer BC film synthesized in different carbon sources (sugarcane molasses, syrup, and fructose) had the ability to retain and slowly release ceftriaxone, an antimicrobial drug (Lazarini et al., 2016).

The 3D network structure of BC is the basis for antibiotic to be contained and released continuously (Buruaga-Ramiro et al., 2020). The BC-TCH composite membrane loading TCH has good antibacterial activity, biocompatibility and controlled release of TCH (Shao et al., 2016a; Figure 4B). Amoxicillin is easy to degrade and then lose its antibacterial activity, so it is very important to limit the release of amoxicillin. Grafting antibiotics onto modified BC is also a commonly used method. Ye et al. (2018) designed a new biocompatible sponge with excellent antibacterial properties by grafting amoxicillin onto regenerated bacterial cellulose (RBC), and the grafted RBC enhanced the antibacterial activity against fungi, Gram-negative and Gram-positive bacteria, which had great potential in clinical application of wound dressings.

### Combination With Inorganic Antimicrobials

Due to the overuse of antibiotics, many bacteria have developed resistance to antibiotics, which greatly reduces the antibacterial effect (Kuehn, 2018; Durand et al., 2019; Subramaniam and Girish, 2020). For the sake of finding new antibacterial agents that are not easy to produce drug resistance and have excellent antibacterial effect to replace antibiotics, people have focused on inorganic antibacterial agents, including various metal/metal oxide nanoparticles, carbon nanomaterials and nanosilicate materials. The combination of inorganic antibacterial agents and BC has always been a research hotspot, particularly nano antibacterial materials composed of metal/metal oxides and BC (Table 2). Dipping, reduction, deposition are commonly used composite methods, the choice of composite methods depends on different situations.

**TABLE 2:** BC-based metal/metal oxide nano-antibacterial composites.

**Metal/metal oxide**	**Preparation methods**	**Bacterial species**	**Mechanism**	**References**
Ag	*In situ* reduction, dipping or photodeposition	*S. aureus* and *E. coli*	Ag^+^ release inhibits cell growth, disrupts cell membrane and prevents DNA replication and transcription, or the generation of ROS destroys cell structure and function	Le Ouay and Stellacci, 2015; Mohite and Patil, 2016; Pal et al., 2017; Sukhorukova et al., 2017; Yang et al., 2017; Moniri et al., 2018b; Tabaii and Emtiazi, 2018; Wu C. -N. et al., 2018; Chatchawanwirote et al., 2019
Au	Dipping	*E. coli*, *MDR E. coli*, *P. aeruginosa* and *MDR P. aeruginosa*	Light excitation produces local heat to destroy cell structure, and Au NPs combine with ribosome subunits to prevent protein synthesis	Cui et al., 2012; Li et al., 2017; Nisar et al., 2019
Cu	Dipping or *in situ* reduction	*S. aureus*, *B. subtilis*, *C. albicans*, *E. coli* and *P. aeruginosa*	Cu^+^ release destroys cell membranes and damages DNA and cell enzyme	Bogdanovic et al., 2014; Shao et al., 2016c; He et al., 2018
TiO_2_	Dipping	*S. aureus* and *E. coli*	Photocatalytic generation of ROS to sterilize	Khalid et al., 2017b; Pathakoti et al., 2019
ZnO	*In situ* deposition or electrochemical deposition	*S. aureus* and *E. coli*	Zn^+^ release and photocatalytic generation of ROS to sterilize	Janpetch et al., 2016; Khalid et al., 2017a; Joe et al., 2018; Luo et al., 2020
Fe_3_O_4_	*In situ* deposition	*S. aureus*, *S. epidermidis* and *P. aeruginosa*	Magnetic thermotherapy produces local heat for sterilization	Raval et al., 2017; Moniri et al., 2018a

#### Metal/Metal Oxide Nanoparticles

**FIGURE 5:**
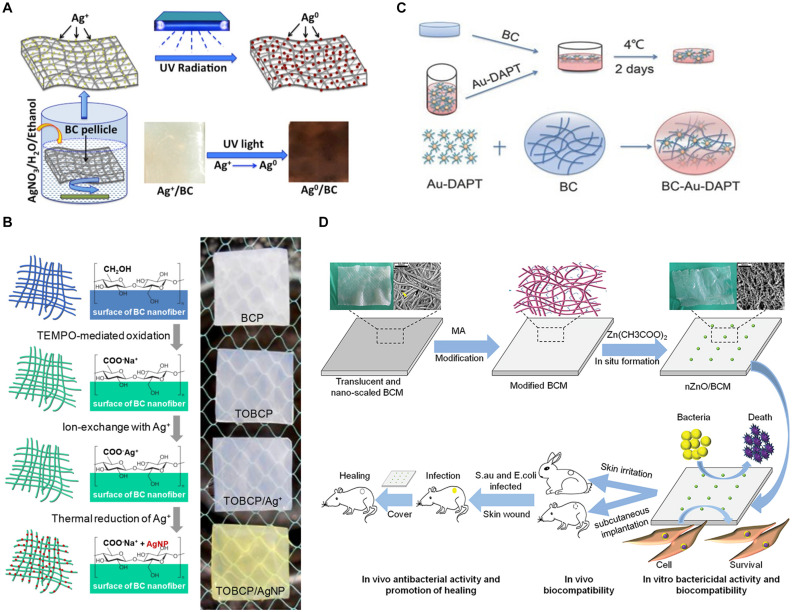
**(A)** Ag/BC films prepared by photochemical deposition (Pal et al., 2017). **(B)** TOBCP/Ag NPs films prepared by thermal reduction method (Wu C. -N. et al., 2018). **(C)** BC-Au- DAPT nanocomposites prepared by solution dipping method (Li et al., 2017). **(D)** nZnO/BCM composites prepared by *in situ* deposition method (Luo et al., 2020).

Silver nanoparticles (Ag NPs) have attracted much attention due to their broad-spectrum antibacterial properties. A great deal of research has shown that these nanoparticles can kill bacteria, but the antibacterial mechanism of Ag NPs is still unclear. Some researchers believe that when silver ion (Ag^+^) released from Ag NPs reaches a certain concentration range it will inhibit the growth of bacteria, destroy cell membrane and prevent DNA replication and transcription. In addition, silver or silver ions can promote the production of intracellular ROS, thus destroying the structure and function of bacterial cells (Le Ouay and Stellacci, 2015; Sukhorukova et al., 2017). BC can be combined with Ag NPs in different ways to obtain antibacterial BC-based materials. The aldehyde or carboxyl groups on BC are usually obtained through oxidation with oxidants, such as sodium periodate and 2,2,6,6-tetramethylpiperidinyloxy (TEMPO). Sodium periodate can selectively oxidize hydroxyl groups on C6 position of BC to form aldehyde group. Through using more sodium periodate and longer oxidation time, oxidized nanocellulose with higher aldehyde group content can be obtained (Sirvio et al., 2011). TEMPO can link on the surface of nanocellulose under aqueous condition and convert the hydroxyl group at C6 position into aldehyde or carboxyl functional groups (Cao et al., 2012). Wu C. -N. et al. (2018) prepared TEMPO-oxidized BC film (TOBCP), which then reacted in AgNO_3_ solution. Ag NPs with a diameter of 16.5 nm were synthesized on the surface of TOBCP nanofibers by thermal reduction method without reducing agent (Wu C. -N. et al., 2018; Figure 5A). The prepared TOBCP/Ag NPs composites have good biocompatibility and high antibacterial activity against *E. coli* and *S. aureus* to be a promising wound dressing. It is also a common way to load Ag NPs on BC by dipping method (Mohite and Patil, 2016; Yang et al., 2017; Moniri et al., 2018b; Chatchawanwirote et al., 2019). For example, in the presence of sodium tripolyphosphate, using AgNO_3_ solution as the precursor and BC film as the template, transparent Ag NPs/BC films were prepared though *in situ* dipping method, whose antibacterial rates to *E. coli* and *S. aureus* were 100% and 99.99%, respectively (Tabaii and Emtiazi, 2018). Because the films were transparent, the wound could be observed continuously without removing the dressing. Furthermore, ultraviolet irradiation can also be utilized for Ag NPs deposition on the BC gel network and Ag NPs combine with the cellulose fiber surface (Pal et al., 2017; Figure 5B). The composite has good antibacterial properties, and Ag/BC film has no obvious silver release after long time immersion.

Due to the potential cytotoxicity of Ag NPs (Mijnendonckx et al., 2013; Hu et al., 2015; Tian et al., 2020), people began to pay attention to other noble metal nanoparticles, such as gold nanoparticles (Au NPs), copper nanoparticles (Cu NPs) and so on. Au NPs have many unique properties, like low toxicity, photothermal effect, large specific surface area, multifunctional surface modification and multivalent effect, etc (Xianyu et al., 2014; Connor and Broome, 2018). For the excellent photothermal effect of Au NPs, local heat is generated under laser irradiation, thus destroying the cell structure. In addition, Au NPs have been proved to bind to ribosomal subunits, which prevent the successful binding of ribosomes and tRNA, resulting in the failed synthesis of protein in bacterial cells (Cui et al., 2012; Nisar et al., 2019). The BC-Au-DAPT nanocomposites prepared by 4,6-diamino-2-pyrimidinethiol (DAPT) and BC have good physicochemical properties and better germicidal efficacy of Gram-negative bacteria than most antibiotics (Figure 5C). It can be used as dressing for the treatment of bacterial infection wounds (Li et al., 2017). In addition, Cu NPs can destroy bacterial cell membrane and damage DNA and cell enzyme via Cu ion (Cu^+^) release (Bogdanovic et al., 2014). A series of RBC membranes loaded with Cu NPs were prepared by Shao et al. (2016c), and the antibacterial activities of RBC-Cu membranes against *S. aureus*, *Bacillus subtilis*, *Candida albicans*, *E. coli*, and *P. aeruginosa* were affected by the content of Cu NPs. *In situ* chemical reduction method was used to prepare BC-Cu NPs films, which showed high long-term antibacterial activity against *S. aureus* and *E. coli* and maintained the continuous release of Cu^+^ after immersion in deionized water for 90 days (He et al., 2018). These results indicate that the antibacterial activity of wound dressing materials can be obtained by loading noble metal nanoparticles such as Ag NPs, Au NPs, and Cu NPs into BC membrane. The 3D network structure and high porosity of BC are conducive to the slow release of metal nanoparticles in composite materials, thus prolongs the antibacterial activity time.

Except noble metal nanoparticles, many metal oxide nanoparticles also have fantastic antibacterial properties, which have attracted the attention of researchers. Titanium dioxide (TiO_2_), zinc oxide (ZnO), ferroferric oxide (Fe_3_O_4_) and other metal oxide nanoparticles were compounded with BC to prepare dressings with antibacterial activity and promoting wound healing. TiO_2_ NPs have photocatalytic activity, which can produce ROS under sunlight to kill bacteria (Pathakoti et al., 2019). BC-TiO_2_ nanocomposites have certain antibacterial activity against *E. coli* and *S. aureus*, good healing potential, faster speed of regenerative epithelialization and ability to accelerate wound contraction (Khalid et al., 2017b). ZnO NPs can kill bacteria through Zn ion (Zn^2+^) release or ROS produced by photocatalysis (Joe et al., 2018). Based on maleic anhydride (MA) modified BC (BCM), the nZnO/BCM biocomposites were prepared using a simple and environmentally friendly method (Figure 5D), which not only showed excellent antibacterial activity against *S. aureus* and *E. coli*, but also prevented bacterial infection and promoted wound healing by accelerating reepithelization and wound contraction (Luo et al., 2020). Janpetch et al. (2016) synthesized ZnO NPs successfully by liquid-phase plasma discharge method without reducing agent, and deposited ZnO NPs into BC film with BC as template. The BC-ZnO composite showed strong antibacterial activity against *S. aureus* and *E. coli* to be used as antibacterial material in wound dressing. On the other hand, ZnO NPs can be directly impregnated into BC to obtain the composite with certain antibacterial activity and healing activity (Khalid et al., 2017a). In addition, Fe_3_O_4_ NPs, as a kind of magnetic nanoparticles, can generate local heat through magnetic thermotherapy to achieve sterilization (Raval et al., 2017). Moniri et al. (2018a) reported the formation and immobilization of spherical magnetic Fe_3_O_4_ NPs (15–30 nm), and BNC/Fe_3_O_4_ nanocomposites with non-toxic have nice antibacterial activity against *S. aureus*, *Staphylococcus epidermidis*, and *P. aeruginosa*. The composites also can increase the expression of other effective genes by reducing the expression of microRNA, thus leading to wound healing.

#### Carbon Nanomaterials

**FIGURE 6:**
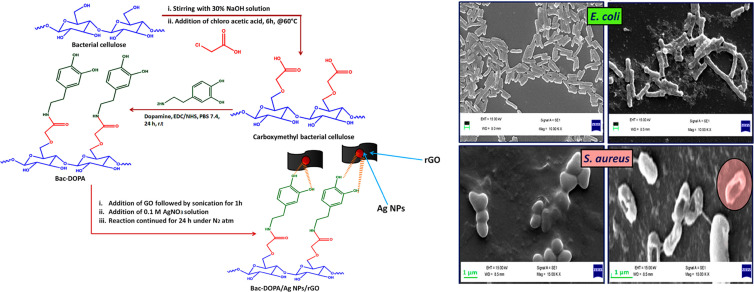
Preparation process and antibacterial effect against *E. coli* and *S. aureus* of BC-DOPA/rGO/Ag NPs composite (Khamrai et al., 2019).

Graphene oxide (GO) is a potential wound dressing material with good biocompatibility and mechanical properties. Moreover, GO has high antibacterial activity against Gram-negative bacteria and Gram-positive bacteria. GO sheets can destroy the phospholipid bilayer along the edge of the sheet, that is, physical damage to bacterial cell membrane (Chen et al., 2014). It can also generate ROS under laser excitation or local heat under near-infrared light, so as to achieve antibacterial (Qiu et al., 2018). A new type of chitosan/BC nanofiber composite reinforced by GO nanosheets was developed. The addition of GO nanosheets reduced the average size of the fibers and affected their mechanical properties (Azarniya et al., 2016). Mohammadnejad et al. (2018) optimized the synthesis of GO-Ag nanohybrids by response surface methodology and impregnated them into BC. It was found that the GO-Ag nanohybrids exhibited synergetic enhanced antibacterial activity against *E. coli* and *S. aureus* at a relatively low dose. In another way, the BNC/polyacrylic acid/GO composite hydrogels were successfully synthesized by electron beam irradiation, and with the increase of GO concentration in hydrogels, their hardness and biocompatibility decreased, thus ensuring the durability and ease of wound dressings removal (Chen et al., 2019). BC was modified by amidation reaction between carboxylated BC and dopamine (DOPA). The BC-DOPA/rGO/Ag NPs composite membrane was prepared through the free hydroxyl group of BC-DOPA, and the composite membrane had antibacterial effect on Gram-positive and Gram-negative bacteria and accelerated the wound healing process because of rGO and Ag NPs (Khamrai et al., 2019; Figure 6).

Furthermore, a series of new BC/C-60 composites were prepared via *in situ* dehydration-rehydration method, which had high ROS production capacity under light conditions and strong antibacterial activity against *E. coli* and *S. aureus*, and could be used as multifunctional wound dressings (Chu et al., 2018). BC has the ability to carry and transfer drugs to assist wound healing. It has been studied that carbon quantum dots-TiO_2_ (CQD-TiO_2_) nanoparticles were added into BC as antibacterial agents, and the BC/CQD-TiO_2_ nanostructure had good antibacterial properties and tensile strength, which provided an alternative method for making antibacterial wound dressings (Malmir et al., 2020).

#### Nanosilicates-Based Materials

Nanosilicates, such as montmorillonite (MMT), are composed of two silica tetrahedral sheets and a central octahedral alumina sheet, plate-shaped particles, and have antibacterial activity to clean and protect the skin, so that they have been widely used in the medical field. The BC/MMT membrane has excellent antibacterial activity against *E. coli* and *S. aureus* and potential therapeutic value in wound healing and tissue regeneration (Ul-Islam et al., 2013). Wasim et al. combined the wound healing characteristics of BC with the antibacterial activity of modified MMT (Cu-MMT, Na-MMT, and Ca-MMT) to obtain modified MMTs-BC nanocomposites that show enhanced wound healing activity and promoted functions of tissue regeneration, reepithelization, healthy granulation, and angiogenesis (Sajjad et al., 2019).

### Combination With Organic Antimicrobials

Considering that there are some noble metal/metal oxide nanoparticles and carbon materials whose residues after use will pollute the environment to a certain extent (Tian et al., 2019), or cause long-term potential toxicity problems in the body and so on (Baker et al., 2014; Vimbela et al., 2017), people are looking for safer antibacterial materials with the least negative impact to the environment and human body. Organic antibacterial agents, including natural polymers (such as chitosan, curcumin, amino acids) and synthetic polymers (such as polyhexamethylene biguanidine), can be combined with BC to prepare antibacterial wound dressings. Among them, natural polymers have attracted more and more attention due to their excellent biocompatibility and degradability. The methods of combining BC with organic antimicrobials include immersion, physical blending, *in situ* synthesis, chemical fixation, electrostatic self-assembly and so on.

#### Natural Polymers

**FIGURE 7:**
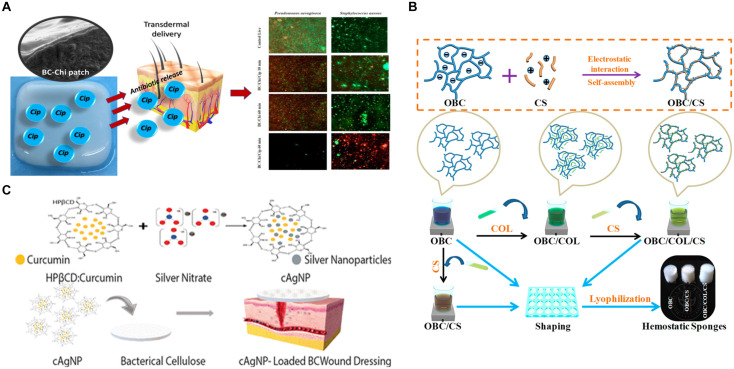
**(A)** BC-Chi-CIP membrane for CIP controlled release with synergistic antibacterial effect of Chi and CIP (Cacicedo et al., 2020). **(B)** The preparation scheme of OBC/COL/CS hemostatic sponge with CS as antibacterial agent (Yuan et al., 2020). **(C)** Schematic representation of the preparation of cAgNP loaded BC-based wound dressing with synergistic antibacterial effect of curcumin and Ag NPs (Gupta et al., 2020).

Chitosan (CS) is considered as a natural antibacterial compound, which can resist a variety of microorganisms, including bacteria, yeast, and mold (Li and Zhuang, 2020; Li et al., 2020). Low molecular weight CS can pass through the bacterial cell membrane and combine with negatively charged nucleic acid, while high molecular weight CS can adhere to the cell membrane and form a membrane on the surface of bacteria, thus hindering the transportation of nutrients (Li J. et al., 2016; Liu et al., 2020). A highly efficient dynamic culture method which retain nanofiber structure can be used to prepare CS/BC nanofiber hydrogel with high mechanical properties and great antibacterial activity against *E. coli* and *S. aureus* (Zhang et al., 2016). In addition, biopolymer blends based on BC membrane modified by low molecular weight chitosan (Chi) were developed for the controlled release of ciprofloxacin (CIP), and Chi had synergistic effect on CIP antibacterial activity (Cacicedo et al., 2020; Figure 7A). A novel hemostatic nanocomposite (OBC/COL/CS) was prepared by coupling oxidized bacterial cellulose (OBC), CS and collagen (COL) (Yuan et al., 2020; Figure 7B). In the electrostatic self-assembly process of OBC and CS, COL was skillfully attached as a functional component by the electrostatic adsorption between cationic CS and anionic OBC. The introduction of COL was expected to enhance hemostasis and promote wound healing, while CS provided antibacterial effect, so the composite material had rapid and efficient hemostatic effect and good antibacterial property.

Curcumin is a natural polyphenol compound, also a hydrophobic drug, with antibacterial activity and wound healing ability, so it is considered as a wound healing agent. Moreover, it is a natural reducing agent, which can be used for green synthesis of nanoparticles. The microencapsulation of curcumin in cyclodextrin (CD) has overcome the hydrophobicity of curcumin (Gupta et al., 2019). It has been studied that the compounds (cAgNP) containing Ag NPs were prepared with curcumin and hydroxypropyl-β-CD and then were loaded into BC hydrogels with moist wound healing properties. The new dressing had not only high biocompatibility but also antibacterial activity to three common pathogenic bacteria in wound infections, *S. aureus*, *P. aeruginosa*, and *Candida* (Gupta et al., 2020; Figure 7C). Hydroxypropyl-β-CD enhanced the water solubility of curcumin and allowed it to be loaded in BC hydrogels, and BC hydrogels could be used for wound treatment, which also had good blood compatibility, cytocompatibility, anti-staphylococci, and antioxidant capacity (Gupta et al., 2019). Besides, a self-healing polyelectrolyte membrane was produced by crosslinking cationic charged CS with anionic modified BC, and the membrane could deliver curcumin (Khamrai et al., 2017). In another way, curcumin was encapsulated in pluronic and prepared into granules by extrusion method, and then the granules were coated with CS, which finally were successfully fixed on the surface of mBNC by EDC/NHC chemical method. The particles attached to the surface of mBNC could control the release of curcumin in a sustained and good way, which made the potential wound dressing have prolonged bioactivity (Rojewska et al., 2017).

In addition, lignin and its derived compounds also have antibacterial properties. In order to develop a highly effective antibacterial dressing for treating chronic wounds, a composite hydrogel (BC-DHP) of BC and lignin dehydrogenate polymer (DHP) was designed, and the BC-DHP hydrogel could release DHP oligomer with antibacterial activity (Zmejkoski et al., 2018).

#### Bioactive Substances

Lysine, tyrosine, arginine and other bioactive substances can promote cell proliferation and collagen deposition related to skin growth, thus accelerating wound healing, and some amino acids can also obtain antibacterial activity after modification. For the sake of exploring the practical application value of BC and developing new wound dressings, a series of BC/PILs composite membranes with antibacterial activity were synthesized by *in situ* synthesis method (He et al., 2020). Polymer ionic liquids (PILs) were formed by choline and different amino acids. Several BC/PILs membranes showed good biocompatibility and high antibacterial activity against Gram-positive and Gram-negative bacteria and fungi. ε-poly-L-lysine (ε-PLL) is a kind of non-toxic biopolymer with broad-spectrum antibacterial activity, and the ε-PLL functionalized BC can be used in wound dressing (Fursatz et al., 2018). Tyrosine (Tyr) is a natural amino acid, which can form non-covalent cation-pi interaction with positively charged ethylenediamine (EDA). A novel compound [EDA][DLA-Tyr] was synthesized by simple coupling reaction based on the dilinoleic acid (DLA) and Tyr. BC impregnated with [EDA][DLA-Tyr] showed strong and long-term antibacterial activity against *S. aureus* and *S. epidermidis*, as a promising new wound dressing (Zywicka et al., 2018).

#### Synthetic Compounds

**FIGURE 8:**
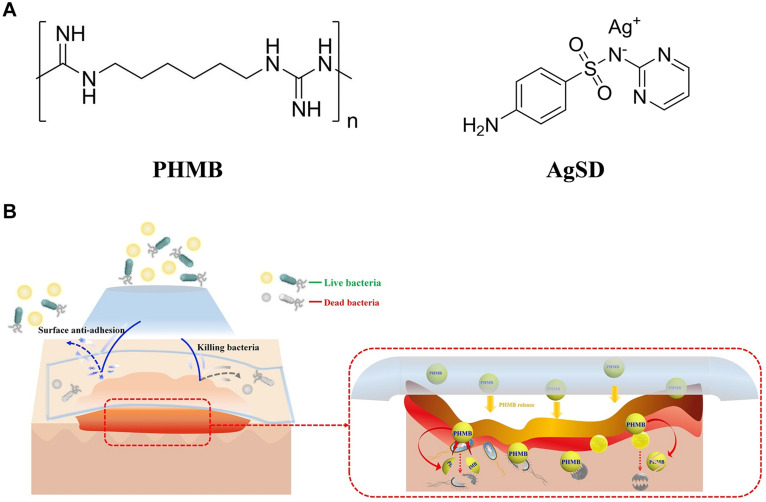
**(A)** Molecular formula of fungicides PHMB and AgSD. **(B)** Multifunctional antibacterial effect of PHMB-PBC composites (Wang et al., 2019).

Polyhexamethylene biguanide (PHMB) is a fungicide (Figure 8A), which has antibacterial effect on Gram-positive bacteria and Gram-negative bacteria. It has been studied that PHMB was mixed with BNC to improve the antibacterial effect of BC-based wound dressing (De Mattos et al., 2019). Napavichayanun et al. (2016) tried to develop a new type of BNC dressing, which was composed of PHMB as an antibacterial agent and sericin as a component for promoting wound healing. The antibacterial PHMB component played a role in reducing infection and inflammatory reaction, so the composite could be used as a safe and effective wound dressing material. The physical and biological properties of BC dressing were improved by adding silk fibroin (SS), PHMB and glycerin, because the dehydration rate of BC dressing decreased with the addition of glycerol to prevent adhesion to the wound, and SS promoted the formation of collagen and tissue in wound surface (Napavichayanun et al., 2018). Another new composite material (PHMB-PBC) composed of BC, polyethylene glycol and PHMB also has good physical and chemical properties, especially transparency, water holding capacity, flexibility and anti-adhesion force (Wang et al., 2019). Moreover, the composite has good biocompatibility and strong antibacterial activity, which can effectively promote the healing and regeneration of rat skin wounds (Figure 8B). In addition, silver sulfadiazine (AgSD) is also an antibacterial agent widely used in local burn management (Figure 8A), which has a wide range of antibacterial and antifungal effects, and its mechanism of action is related to the ionization and release of Ag^+^ (Dellera et al., 2014). When the ions are released too much, cytotoxicity may appear. Combination with BC can reduce the possible cytotoxicity (Liu et al., 2019), enhance the antibacterial activity and promote wound healing. For example, AgSD can be impregnated into BC matrix, and the obtained composite has pH sensitive controlled-release behavior and good antibacterial activity against *S. aureus* and *C. albicans* (Shao et al., 2016b).

## Problems and Challenges

Through the summary of the preparation and research progress of BC-based antibacterial wound dressing, we can find that there are still many problems in the composite materials, including the preparation methods and antibacterial activity. The commonly used method of adding antibiotics has been widely used in clinical practice. BC with 3D network structure can help retain and slowly release antibiotics, but the problem of resistance to antibiotics is still under study until now. People are willing to find a new therapy to fight against drug-resistant bacteria, for example, there are antibodies, probiotics and vaccines in the early stage (Czaplewski et al., 2016). Later, it is found that antibacterial nanomaterials are expected to replace antibiotics, however, nanoparticles have the disadvantages of easy aggregation, uncontrollable ion release trend and potential cytotoxicity, which limit their application (Sulaeva et al., 2015). Therefore, it is worth studying to prevent the aggregation of nanoparticles, facilitate immobilization and controlled release of nanoparticles, and improve sterilization efficiency while reducing the use of nanoparticles. Due to a large number of active functional groups, BC and its modified products can be used as the green reducing agent, stabilizer, template or immobilization material for nanoparticles, which is beneficial to reduce the agglomeration of nanoparticles and control the release rate. People have been pursuing the development of antibacterial materials without drug resistance, cytotoxicity and pollution, but long-term controllable antibacterial activity. Although natural organic antimicrobials have wide sources and good biodegradability, they have some problems such as inadequate antibacterial properties or unstable antibacterial activities. On the contrary, synthetic organic antimicrobials have some limitations in biodegradability. Therefore, there is no defect-free BC-based wound dressings material, and developing multifunctional BC-based composites is a major direction of future research. The materials compounded with BC should not only give antibacterial activity, but also improve other physical and chemical properties of composites to help wound healing.

Low yield, high capital investment requirements, and associated high operating costs present major economic constraints to the commercialization of BC production. How to reduce the energy consumption and cost of material preparation is still a big challenge. The BC potential in advanced material applications are hindered by a limited knowledge of optimal BC production conditions, efficient process scale-up, separation methods, and purification methods (Reiniati et al., 2017), so BC-based wound dressings have not been widely used in clinic. By utilizing low-cost substrates and wastes from agro-industry, breweries, food producers, and municipalities, it is possible to devise an economically feasible biotechnological process for BC production, but its high selling cost still restrict BC to high-value markets (Ul-Islam et al., 2020). In conclusion, BC-based wound dressings should be developed in the direction of low cost, simple process, green environmental protection, safety, no potential toxicity, excellent physical and chemical properties, and sustainably controlled sterilization.

## Conclusion

Wound repair is a physiological process affected by many factors, and its complexity often leads to some uncertainties, such as slow wound healing, secondary infection and inflammation. BC-based materials are a kind of promising wound dressing materials with many excellent properties that are beneficial to wound healing, such as providing a moist wound environment, good biocompatibility, and stable mechanical properties. However, they lack antibacterial properties. The antibacterial activity of wound dressings plays an important role in anti-infection and promoting wound healing during wound treatment. In order to obtain antibacterial activity and accelerate wound healing, different kinds of antibacterial substances were compounded with BC, including metal or metal oxide nanoparticles, carbon nanomaterials, nanosilicates, antibiotics, natural polymers, bioactive substances, and synthetic polymers. Wound dressings consisted of BC and antibiotics are most frequently used with potential clinical application value, while BC combined with metal or metal oxide nanoparticles have great antibacterial effect. Moreover, wound dressings possess excellent biocompatibility and degradability without potential toxicity and pollution when they are made of BC and natural polymers. BC-based antibacterial materials obtained with these methods have significant antibacterial activity and the potential to reduce infection and promote wound healing. Nevertheless, there are also some problems, such as drug resistance caused by abuse of antibiotics, environmental pollution, potential toxicity of metal/metal oxides, and unstable antibacterial activity of natural antibacterial polymers. Overcoming these problems is also an important direction for future development. Furthermore, organic/inorganic hybrid materials, natural/synthetic hybrid materials and emerging nanomaterials are all research hotspots, as they can be used to construct new antibacterial wound dressings by combination with BC.

## Author Contributions

LZ and XW conceived the scope of this work. LZ drafted the manuscript. SL, JL, and XW involved in revising and editing the manuscript. All authors approved the submitted version and agreed both to be personally accountable for the author’s own contributions and to ensure that questions related to the accuracy or integrity of any part of the work.

## Conflict of Interest

The authors declare that the research was conducted in the absence of any commercial or financial relationships that could be construed as a potential conflict of interest.
